# 1895. Omadacycline for the Treatment of Non-Pulmonary Mycobacterial Infections: a Single-Center Retrospective Review

**DOI:** 10.1093/ofid/ofad500.1723

**Published:** 2023-11-27

**Authors:** Edmund Shen, John Albin, Kristen Hysell, Rocio M Hurtado

**Affiliations:** Harvard Medical School, Boston, Massachusetts; Massachusetts General Hospital, Boston, MA; Massachusetts General Hospital, Boston, MA; Massachusetts General Hospital, Boston, MA

## Abstract

**Background:**

Infections due to rapidly growing mycobacteria (RGM) such as *M. abscessus*, *M. chelonae* and *M. fortuitum* typically require long and toxic regimens, with complex resistance patterns and limited oral options further producing suboptimal efficacy. Since its approval in 2018, however, the tetracycline derivative omadacycline has established new options in antibiotic management. We report here clinical experience with the use of omadacycline for non-pulmonary RGM infections within the Mass General Brigham (MGB) system.

**Methods:**

The study was approved by the MGB Institutional Review Board (2022P001578). Electronic ambulatory notes in the MGB Research Patient Data Registry (RPDR) from 2012-present were filtered by the term “omadacycline,” and manual chart review was conducted for individuals treated for non-pulmonary RGM infections.

Flow diagram of chart review procedure for identification of non-pulmonary RGM patients

**Figure:**
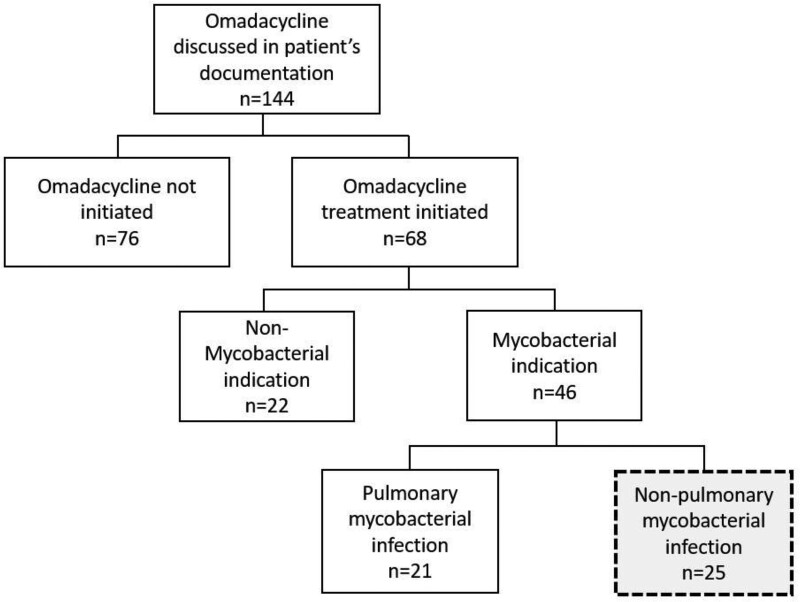

**Results:**

Chart review revealed 25 patients with omadacycline-treated non-pulmonary RGM infections. The median age was 50, 52% female and 48% male. In total, 16 were infected with M. abscessus (64%), 8 with M. chelonae (32%), and 1 with M. immunogenum. 18 cases were skin and soft tissue infections (72%), 4 cases were bone and joint infections (16%), and 3 cases were disseminated infections (12%). Most patients (88%) received omadacycline as part of a step-down or a salvage regimen; 3 (12%) received omadacycline in initial treatment. 17 patients (68%) had completed regimens as of April 2023. Among these cases, the median duration of omadacycline use was 5 months (range=0.25-16). Patients within this cohort generally experienced clinical improvement. Adverse effects–most commonly nausea–were noted in 6 (24%) cases. No patients were discontinued due to the severity of these effects, though one regimen was temporarily paused due to hyperlipasemia.

Non-pulmonary RGM patient cohort demographics, Mycobacterial species, and site of infection.

**Figure:**
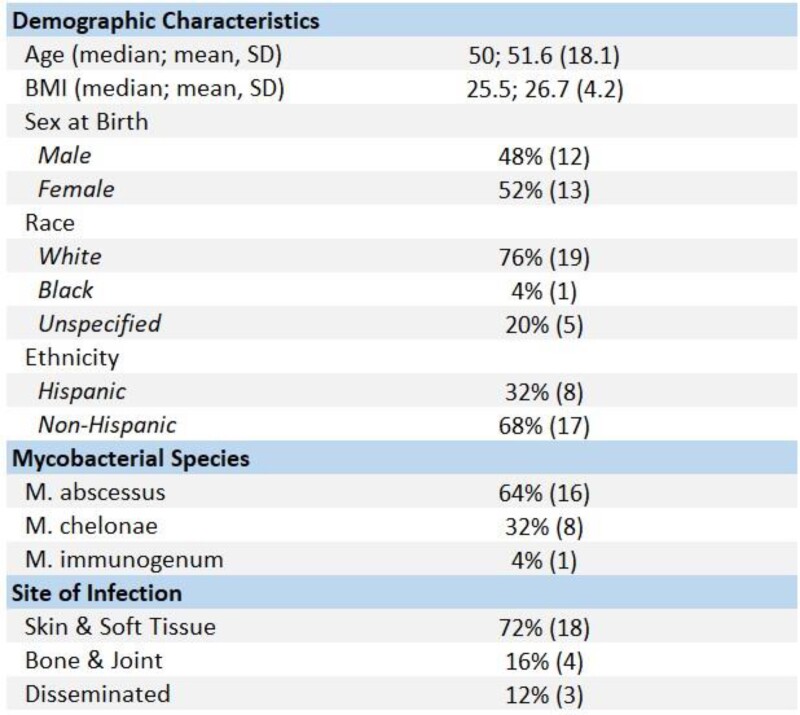

**Conclusion:**

Omadacycline is an increasingly common component of the treatment of RGM infections, with use in multidrug regimens appearing generally effective and well-tolerated. Limitations of this study include its retrospective nature and evaluation within a single health system. To our knowledge, however, this is the largest series on omadacycline in RGM infections reported to-date. This analysis provides support for the further evaluation of omadacycline outcomes among patients with RGM infections.

**Disclosures:**

**All Authors**: No reported disclosures

